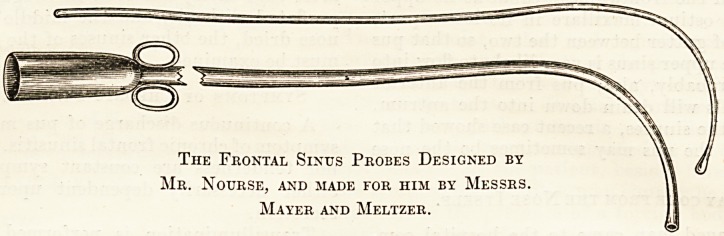# Chronic Suppuration in the Nasal Accessory Sinuses and Its Treatment

**Published:** 1906-08-18

**Authors:** Wm. J. Chichele-Nourse

**Affiliations:** Surgeon to the Central London Throat, Nose, and Ear Hospital; late President of the British Laryngological, Rhinological, and Otological Association.


### Hospital Clinics.

### CHRONIC SUPPURATION IN THE NASAL ACCESSORY SINUSES AND ITS TREATMENT.

Two Lectures by Wm. J. Chichele-Nourse, F.R.C.S.(Edin.), Surgeon to the Central London Throat, Nose, and Ear Hospital; late President of the British Laryngological, Rhinological, and Otological Association.

These lectures were specially reported for the columns of The Hospital to illustrate the nature of the graduate teaching given at this important special centre. The notes have very kindly been revised by the lecturer.

### The Grouping of the Sinuses.

For clinical purposes, the accessory sinuses of the nose may be divided into two groups, according to the position of their ostia. The maxillary antrum, the frontal sinus, and the anterior ethmoidal cells, having their ostia opening into the middle meatus of the nose, form an anterior group. The sphenoidal sinus and the posterior ethmoidal cells, opening into the superior meatus, form a posterior group.

In each group the ostia lie very close together. Those of the anterior group are situated in the hiatus semilunaris under cover of the anterior end of the middle turbinal body; that of the frontal sinus above, the ostium maxillare below, and the openings of the ethmoid cells close by the others. Sometimes the antrum has an accessory ostium, opening in the middle meatus much further back.

The ostium of the sphenoidal sinus is situated high up and far back in the posterior wall of the nose, and those of the posterior ethmoidal cells in the spheno-ethmoidal recess.

### The Sinuses in Health.

In health, the lining of the sinuses consists of a very thin mucous membrane, blended inseparably with the periosteum, the whole forming a thin layer closely adherent to the bony wall beneath. The blood-supply is scanty, and the walls of the sinuses are almost destitute of secretory glands.

### The Sinuses in Disease.

The sinuses are liable to several diseases:—

Acute sinusitis, the result either of the extension of an acute catarrh from the nose, or of infection during one of the specific fevers, such as typhoid, scarlatina, or (especially) influenza. Chronic sinusitis, either the sequel of an unhealed acute catarrh or, possibly, originating *de novo*.

Simple Empyema: Cystic disease or mucocele, and benign and malignant tumours. Besides these, the sinuses are liable to be invaded by malignant disease or by gummata arising in neighbouring parts, or their bony walls may become necrosed or carious.

### Chronic Sinusitis.

The key to the study of diseases of the sinuses is chronic sinusitis. To this part of the subject, therefore, as the time is limited, it is proposed to confine the following observations:—

In chronic sinusitis the muco-periosteum becomes enormously thickened, vascular, and polypoid, and secretes pus, and occasionally small abscesses have been found embedded in it.

The local signs are often obscure, so that the diagnosis is not always easy; but the patient may suffer from pharyngeal or laryngeal irritation, gastric or bronchial disturbance, or general malaise without obvious reason, but really caused by the absorption of septic pus flowing unobserved into the naso-pharynx.

### The Diagnosis.

The best mode of proceeding in the diagnosis is to take the sinuses in order, beginning with those of the anterior group, and to deal with each one by exclusion before passing to the next. It must be remembered that sinusitis is but rarely isolated; hence the discovery that one sinus is affected must not deter the surgeon from investigating the condition of the others. Frontal sinusitis is often accompanied by antrum disease, and an affection of either of these sinuses is generally associated with suppuration in the neighbouring ethmoidal cells. Again, sphenoidal sinusitis is usually linked with disease in the posterior ethmoidal cells, and *vice versa*. Occasionally all the sinuses on one or both sides are affected, when the condition is called pansinusitis.

Lermoyez Classification of the Signs.

Lermoyez classified the signs of chronic suppuration in the accessory sinuses as presumptive, probable, and certain. The following table is based upon this division:—

PRESUMPTIVE SIGNS.

1. Subjective fœtor.
2. Pain, relieved by liberation of discharge.
3. Unilateral flow of pus from the nose.
4. Polypi or polypoid hypertrophies in the middle meatus.

PROBABLE SIGNS.

Antrum:
Pus in the middle meatus.
Discharge intermittent, increased on bending forward or on bending the head to the opposite side.
Infra-orbital pain.
Opacity on transillumination.
Swelling, redness, and tenderness in the canine fossa.
Swelling of cheek (rare).
Bulging of nasal wall of antrum.
N.B.—Distinguish from simple empyema.
(*a*) Nasal type:
With suppuration in nose, or in other sinuses of anterior group.
(*b*) Dental type:
Upper molar or bicuspid caries.
Tooth tender on percussion.
Verify by
Puncture through nasal wall, perflation, and irrigation.
Sign of capacity (Mahu).
Blood coming through cannula (Lubet-Barbon).
Opacity persists after puncture (Guisez and Guérin).

Frontal Sinus:
The antrum being excluded or emptied—
Pus in middle meatus.
Discharge persistent.
Pain; usually frontal.
Frontal tenderness.
Opacity on transillumination.
Verify by
Cannula and perflation.

Anterior Ethmoidal Cells:
After exclusion of antrum and of frontal sinus—
Pus in middle meatus, often profuse.
Pain; supra-orbital or lachrymal.
Tenderness in the same regions.
Bulging of ethmoid cells into nose.
Asthenopia.
Mental depression.
Granulations, etc., in middle meatus.
Verify by
Use of probe.
Puncture of bulla.

Sphenoidal Sinus:
Pus in superior meatus;
between septum and middle turbinal (anteriorly).
on superior and middle turbinals (posteriorly).
Pain, sometimes occipital.
Ocular disturbance.
Polypi.
Verify by
Cannula and perflation.
Pus from ostium seen.
N.B.—Avoid exploratory puncture.

Posterior Ethmoidal Cells:
Having excluded the sphenoidal sinus by irrigation—
Pus persists.
Bare bone felt.
Polypoid middle turbinal.
Verify by
Effects of treatment.

Practical Significance.

The vagueness of the local symptoms of chronic sinusitis has already been alluded to. The classic signs given in the older text-books are conspicuous by their absence, and what may be called directing signs are often altogether wanting.

The particular value and significance of the "presumptive signs" is that the presence of any of them indicates the need for a systematic examination of the sinuses. It must be understood that they are not pathognomonic—a unilateral discharge from the nose accompanied by an offensive smell observed by the patient, besides being a possible symptom of sinusitis, may equally be due to a rhinolith, to the presence of a foreign body in the nostril, or to syphilitic necrosis.

Pain, relieved by liberation of discharge is strongly suggestive of suppuration, but unfortunately it is not a constant sign. The occurrence of pain in sinus disease may be entirely independent of retention, or it may be altogether absent.

Polypi are almost always associated with suppuration.

Probable Signs.

Commencing with the antrum, the probable signs given in the table need a few words of comment. If pus can be seen in the nose its situation affords some indication of the sinuses probably affected. When the secretion lies between the anterior extremity of the middle turbinal and the outer wall of the nose the suggestion is that its source is in the sinuses of the anterior group. On the other hand, when pus appears between the middle turbinal and the septum, or above the middle turbinal, it most likely comes from the posterior group. If the antrum has an accessory ostium, the whole of its secretion may drain backwards into the naso-pharynx and be quite invisible when the nose is examined from the front.

The ostium of the antrum is situated at a level considerably above the floor, so that secretion may accumulate in that cavity, and will flow out on altering the position of the head.

Transillumination and its Interpretation.

Transillumination is performed by introducing an electric light into the mouth so as to illuminate the soft parts of the face. If the antra are normal, an illuminated crescent appears at the lower edge of the orbit, the pupil of the eye reflects a red glow, and the patient is aware of a sensation of light. These phenomena are best marked in young people and females; they are absent when the antrum is wanting, when its walls are abnormally thick, when it is occupied by a tumour, or when it contains pus.

Chronic Sinusitis or Simple Empyema.

A distinction must be made between chronic sinusitis of the antrum and simple empyema. In sinusitis the lining membrane of the antrum is in a state of disease, and is secreting pus; in simple empyema, on the other hand, the lining of the antrum is healthy, but the cavity becomes a reservoir for pus, which drains into it from elsewhere.

The pus in empyema may have flowed into the antrum through the ostium maxillare from the frontal or the anterior ethmoidal cells. A glance at the anatomical arrangement of the outer wall of the middle meatus will explain one way in which this may occur. The hiatus semilunaris, having the opening of the frontal-nasal canal at its upper end, and the ostium maxillare in its lower part, forms a sort of gutter between the two, so that pus formed in the upper sinus is very likely to flow into the lower; probably, also, pus from the anterior ethmoidal cells will drain down into the antrum. But, besides the sinuses, a recent case showed that the source of the pus may sometimes be the nose itself.

### Pus may come from the Nose Itself.

A middle-aged man came to the hospital complaining of subjective fœtor and discharge from the left nostril; the left antrum was opaque on transillumination, and on puncture and irrigation through the nasal wall proved to contain a quantity of fœtid pus.

Upon further examination, the patient was found to have a breaking-down gumma in the middle meatus, from which the pus drained into the antrum. The ordinary treatment for specific disease, and one or two irrigations of the antrum through Lichtwitz' trocar, cured the disorder.

### Origin may be Dental.

The other source of pus in emypema is dental. A peri-apexial abscess at the root of a carious bicuspid or molar may burst into the floor of the antrum and continue to discharge pus into the cavity until a large quantity has accumulated. Caries of upper teeth, or tenderness of a tooth upon percussion, will create a suspicion of empyema from a dental source.

The presence of pus in the antrum may be verified by puncture with a fine trocar through the inferior meatus, perflation, and irrigation with a clear antiseptic lotion.

### Differential Diagnosis of Sinusitis and Empyema.

The differential diagnosis between sinusitis and empyema is not so easy: the effects of repeated puncture and irrigation, and the presence of foci of suppuration in other sinuses or in the nose, are factors which have to be considered.

Mahu has pointed out that in true chronic sinusitis the mucosa is greatly increased in thickness even during the early stages, so that the capacity of the sinus is considerably reduced. The antrum having been washed out with a fine canula through the inferior meatus, he advises that its capacity should be measured by filling it with lotion, which is then to be withdrawn with a graduated syringe. If the quantity of liquid sucked out in this way is less than 1.5 cubic centimetres, the case is probably one of true sinusitis.

A drop or two of blood sometimes flows from the cannula after puncture: this is thought by Lubet-Barbon to prove that sinusitis is present. Another differentiating sign, suggested by Guisez and Guérin, is that in simple empyema the opacity on transillumination should disappear immediately after washing out the pus, whereas in sinusitis the opacity, being due to the thick vascular lining, persists.

If pus reappears in the middle meatus after it is definitely proved that the antrum is healthy, or after that cavity has been thoroughly washed out, perflated with air, and the middle meatus of the nose dried, the other sinuses of the anterior group must be examined.

### Symptoms of Chronic Frontal Sinusitis.

A continuous discharge of pus may be the only symptom of chronic frontal sinusitis. Neither pain nor tenderness are constant symptoms, nor are either necessarily dependent upon retention of secretion.

Transillumination is performed by placing a hooded lamp in the inner angle of the roof of the orbit. Owing to the occasional absence of the sinus, to the variations in its size and in the thickness of its anterior wall, this test is much less reliable than for the antrum.

The ostium of the frontal sinus is situated at its posterior and lowest point, so that in sinusitis the pus tends to slowly drain away. Permanent retention is not common, but occasionally such cases occur with the formation of an orbital abscess. As a rule the fronto-nasal canal becomes larger than the normal, probably owing to an atrophic condition produced by continuous contact with pus. The result of this is that no large amount of pus accumulates in the sinus at one time, but there is a continuous scanty flow, sometimes so small as to be scarcely noticeable: this is the most usual condition found. Sometimes, however, a granulation or a cluster of polypi may impede the exit of discharge, which then collects in greater quantity; or the lining of the fronto-nasal canal may become inflamed and swelled from some temporary cause, with the same result.

### Verification of Diagnosis.

In order to verify the diagnosis, it has been proposed to distinguish the source of the pus by using small tampons of gauze for damming the ostia of the various sinuses in turn; but, owing to the difficulty in seeing the parts, this is not reliable. The only certain way of proving that the frontal sinus is suppurating (besides opening the sinus) is to pass a suitably curved cannula into it from the infundibulum and to blow or wash out the contents. We can then be quite sure of the source of the pus. In health the fronto-nasal canal is often merely a narrow chink winding between ethmoid cells, and the introduction of even a fine curved probe is difficult and often impossible; but in sinusitis the experience of the author is that it is usually possible to pass a cannula.

The most conveniently shaped instrument is one with a bold curve at the end, forming about one-third of the circumference of a circle, so that the point is directed upwards and forwards during introduction. When the point is engaged in the fronto-nasal canal it slips on by a sliding movement round the circumference of its curve, the handle being gradually depressed, until the point impinges on the front wall of the sinus. The manœuvre must be carried out without any force.

After introduction, the position of the cannula can be estimated by placing the probe parallel to it along the outside of the nose; air is then blown through the cannula, while the middle meatus is under observation. If the sinus contains pus, a few drops will be driven out by the side of the cannula. If necessary, the sinus can be irrigated.

The Frontal Sinus Probes Designed by Mr. Nourse, and made for him by Messrs. Mayer and Meltzer.

Suppuration in the Ethmoidal Labyrinth.

Some of the ethmoidal cells bordering on the fronto-nasal canal are usually involved when the frontal sinus is diseased.

Suppuration in the ethmoidal labyrinth is met with independently of disease in the frontal sinus or the antrum as well as in association with them. It is often accompanied by the formation of polypi and by polypoid degeneration of the nasal lining in the middle meatus; the ethmoid region bulges inwards into the nose, and may cause an apparent duplication of the anterior end of the middle turbinal. One or more of the cells may become greatly dilated at the expense of others, and will then contain quite a large quantity of pus; the discharge is often profuse. Ziem has drawn attention to the ocular symptoms produced by this and other diseased conditions in the nose and accessory sinuses. The diagnosis can be confirmed by using the probe, which will sometimes pass a considerable distance into dilated suppurating cells. Further confirmation can be obtained by the liberation of pus during treatment.

The posterior group of sinuses is situated in an inaccessible region at the back of the nose, and the diagnosis of their diseases is correspondingly difficult.

The Shhenoidal Sinuses and the Symptoms of Suppuration.

The ostium of the sphenoidal sinus is a very small aperture high up in the posterior wall of the nose. In the healthy nose it cannot be seen from the front, being hidden by the middle turbinal body, but in diseased conditions, when the turbinal has been destroyed or atrophied, it becomes visible, and discharge can sometimes be seen coming from it. The ostia of the posterior ethmoidal cells are situated close to the former in the spheno-ethmoidal recess, looking backwards; they are quite out of sight. Pus coming from the sinuses of the posterior group is usually seen from the front lying between the middle turbinal and the septum; and, on posterior rhinoscopy, flakes may be observed adhering to the roof of the naso-pharynx or on the hinder ends of the middle and superior turbinal bodies.

The symptoms of suppuration in this region are often suggestive of commencing atrophic rhinitis; there may be fœtor, a certain amount of wasting, and a tendency to the formation of crusts. Subjective fœtor is commonly complained of. The closeness of the sphenoidal sinus to the optic nerve, the cavernous sinus, and other important intracranial structures and the occasional dehiscence of its bony roof will account for the serious symptoms occasionally observed in cases of suppuration in this cavity, in dealing with which both caution and gentleness must be exercised.

Diagnosis Confirmed.

The diagnosis of suppuration in the sphenoidal sinus may be confirmed by examining the sinus with a probe and perflating or irrigating it by means of a cannula. It may be well to observe that the introduction of instruments into the nose or sinuses should never be attempted except under good illumination by means of a forehead mirror. The probe, which should be nearly straight, and preferably graduated in centimetres, is passed obliquely upwards and backwards between the nasal septum and the middle turbinal body until it touches the posterior wall of the nose. By moving the point slightly the ostium will probably be met with, and the probe will pass on for some distance further. The average depth of the further wall of the sinus touched by the probe is 8.5 centimetres from the lower edge of the nostril. The cannula is passed in the same manner. If by chance the posterior nasal wall is within view, the manipulation is so much easier, and the diagnosis can be confirmed by observation.

When pus persists in the posterior part of the nose after the definite exclusion of the sphenoidal sinus, it is to be presumed that it comes from the posterior ethmoidal cells. This would be verified during surgical treatment.

*(To be continued.)*

## Figures and Tables

**Figure f1:**